# Book Reviews

**Published:** 1826-07

**Authors:** 


### CRITICAL ANALYSES.

Quæ laudanda forent, et quæ culpanda, vicissim

Illa, prius, cretâ; mox hæc, carbone, *notamus*.—PERSIUS.

*An Inquiry concerning that disturbed State of the Vital Functions usually denominated Constitutional Irritation.* By BENJAMIN TRAVERS, F.R.S. senior Surgeon to St. Thomas's Hospital; President of the Hunterian Society of London; Honorary Member of the Royal Medical Society of Edinburgh, and the Medico-Chirurgical Society of Aberdeen; of the Royal Society of Medicine, and the Medical Society of Emulation, of Paris; of the Imperial Medico-Chirurgical Academy of St. Petersburgh; the Medical Society of Stockholm, &c. &c.—8vo. pp. 556. London: Longman and Co. 1826.

THE appearance of another volume by Mr. TRAVERS cannot fail to fix the attention of the professional reader. The works he has previously published have become established in reputation,—have been translated into various languages,—and are every where looked upon as books of authority and reference. But, in addition to the well-earned character of the author, the subject discussed in the volume before us is one of the most interesting and intricate within the range of pathological inquiry: it is only of late, indeed, that it has begun to excite particular attention; and the number of fatal cases that have been recorded during the last few years, forms a striking contrast to the silence of the older writers. This may, we conceive, be fairly attributed to the advanced state of our knowledge of disease generally; a knowledge which now enables us to distinguish many serious morbid affections, formerly unknown or confounded together.

We now proceed, without further preface, to present our readers with as distinct an account of the views of the author before us as the difficulty of the subject, and our restricted limits, permit us to offer. "Since my mind (says Mr. Travers,) was first directed to the practice of my profession, it has been in a particular manner interested by the subject of this treatise. The phrase 'irritation,' although it has no precise idea affixed to it, is in constant use, and appears so to satisfy or to suspend inquiry relative to unforeseen results, as to be little better than an empirical subterfuge.

"My object, therefore, in undertaking this inquiry, was to ascertain with more precision the morbid state indicated by the term 'constitutional irritation;' to investigate the causes most commonly productive of that state, the phenomena by which it is manifested, and the laws by which it is governed; and, from the comprehensive view thus obtained, to derive, if possible, some permanent pathological characters, which might serve as a guide to more correct notions of its nature, and more scientific principles for its treatment." (P. vii.)

Our author does not congratulate himself upon having succeeded in accomplishing his design, and only claims credit for perseverance in the pursuit; justly adding, that the extent and intricacy of the subject may well discourage persons, however well qualified, from the attempt. Mr. Travers adds, that the difficulty is increased by the circumstance "that fatal cases are kept in shadow by modern authors;" and we readily admit that, generally speaking, authors are too apt to publish only those cases which are supposed, from their fortunate termination, to confer lustre upon the narrators: at the same time, we think that, on this particular subject, unusual candour has been evinced.

The Preface concludes with the following passage, from which we regret to find that the author has been unable to complete his design in the present volume:—" Although the size of this volume, independent of the republished documents, has far exceeded my original intention, it leaves the design unfinished. The illustrations of what I have called 'reflected irritation' remain; and, as this department of my subject comprehends the investigation of some of the most important and interesting diseases which fall under the notice of the surgeon, I entertain the hope of presenting, at a future day, another volume to the profession." (P. ix.)

The circumstances at which we have hinted above oblige us to hasten over the preliminary matters more speedily than we should otherwise have been inclined to do, in order that we may dwell more at large upon that portion of the work in which the author's peculiar views and opinions are developed. It may, however, be necessary previously to acquaint the reader that the volume is divided into six chapters, and these again subdivided into sections; the titles of which we shall give as we pursue the course of our analysis.

The first chapter treats of *the influence of constitution in modifying the effects of local injury:* this is subdivided into two sections, the first comprising *Irritability as a principle of Health,* and the second *Morbid Irritability.* There is nothing stated in the first section but what is generally acknowledged. The laws of irritability, as far as we are acquainted with them, are cursorily noticed; and Mr. Travers avows himself a disciple of Mr. Hunter.

In the second section, on *Morbid Irritability*, the author thus defines what he means by the term;

"An irritable mind is one easily excited, and over excited in reference to the occasion, whether to joy or anger, fear or pity. An irritable stomach is nauseated by many ordinary articles of diet. An irritable bladder is continually parting with its secretion before the stimulus of distention can be supposed to act. An irritable heart, if quickened by exertion or strong mental excitement, becomes tremulous and palpitating. An irritable skin breaks into a rash from many slight causes of excitement, both of diet and temperature; a plaster, which is in others only rubefacient, acts upon it like a blister. Irritable fauces are slightly sore with a change of the wind, or exposure for a few seconds to a current of air. And an irritable retina is distressed to dimness by a full light, and, like the mind, disturbed by the surviving representation of transitory impressions." (P. 8.)

He next proceeds to describe *proper* and *sympathetic* irritability: the first he illustrates by the possibility of a man's losing his senses from a shock of the mind, the body discovering no sign of disorder. Thus, a young lady was found playing with the fingers of a skeleton, which had been placed in her bed, and lived to old age, in good bodily health, "but without a glimmer of returning reason." The second form of irritability is familiarly exemplified by the sight of savoury food exciting a flow of saliva, and other examples of this kind, showing that here this property is sympathetic.

Our author next notices the connexion between *sympathy* and *irritability*, and gives us Mr. Hunter's well-known definition of an irritable habit. Examples of this habit are then presented to us in a variety of details, both as they occur in the mind and body. Some of them are curious, but we cannot stop to notice them: among them are some remarkable instances of the effects of high excitement of the mind, such as is produced by the dread of painful operations. Our readers will remember one or two striking examples of this kind in Baron Larrey's Surgical Campaigns.

Among the most interesting portions of this section, we select the concluding passage, not only on account of the good sense contained in it, but also as affording a useful hint to hospital surgeons for the preparation of their patients generally for the greater operations; a point we fear not so much attended to as it deserves.

"Continuous inflammation is most frequent in persons who incur injuries and undergo operations in a state of health. I have known the amputation of a stiff finger, submitted to as a matter of convenience, fatal from this cause. A case occurs to my recollection, of the removal of a small tumor from the scalp of a lady, of which the issue proved disastrous, by inducing erysipelas of the scalp; and I recall other instances of trifling operations, performed in health, in which serious consequences were clearly avoided by imposing a rigorous restraint, and adopting a vigilant plan of treatment. My ingenious friend, Mr. George Young, whose retirement from practice is a subject of general regret, was accustomed to impose restraint before the performance of operations requiring confinement, with excellent effect. Having to extract a cartilage from the knee-joint, he would keep the man, for a week prior to the operation, in the same position as was to be maintained for the week subsequent to it. Thus the irritation of confinement, as well as that of motion, was avoided.

"The following is an illustration of Mr. Young's practice in his own words:—'A healthy carman came under my care with a loose cartilage in the right knee-joint. It had several times occasioned him to fall suddenly, and he was very anxious to submit to an operation to get rid of it. It appeared to me desirable to accustom him, before the operation, to the reduced diet, rest, and restraint, which would be necessary after it. He accordingly kept the house. On the second or third day of his confinement, I put on the roller, and bound on the back splint, exactly as I intended to do after the operation, to keep the limb perfectly steady. This confinement of the limb occasioned a restless night, some fever, a whitish tongue, a quickened pulse, a little headache, spare and high-coloured urine. He was very unwilling to continue the bandage and splint, to which he ascribed (and justly) all his constitutional disturbance, and the utility of which, prior to the operation, he could not at all comprehend. This circumstance, however, forcibly suggested to me the importance of accustoming him to restraint; it was therefore continued. The excitement which it had produced gradually subsided, and, when I found that the bandage no longer occasioned any irritation, I performed the operation. Not one untoward symptom arose, the constitution was not in the least ruffled, and the wound healed by the first intention.'" (P. 36.)

We had nearly forgotten to mention that there is a pretty long note attached to this section, in which the minute dietetic regulations, so much insisted upon by a certain class of practitioners, is rallied with much effect.

Chapter 2d is on the ***Effects of Local Injury on the Constitution,*** and commences thus:

"Irritation is the name given to that state which is produced by an extraordinary excitement of the irritability either of a part or of the system: irritation is, therefore, local or constitutional. The phenomena of irritation are chiefly displayed in the nervous system, and it is thus distinguished pathologically from inflammation, which belongs to the vascular. Their relation is as intimate as that of these systems, of the extraordinary actions of which they are the results. As the causes and degrees of excitement are various, so are the signs and modes of irritation. Inflammation has been sometimes considered a healthy, because a healing action: this, though not a strictly logical, is an intelligible phraseology. In the same sense, the minor degrees of irritation also frequently serve a salutary purpose, and conduce to the preservation of the system. I object to the term 'irritative fever,' as synonymous with irritation, because irritation and fever are, in their nature, as distinct as irritation and inflammation; although their reciprocal affinities are as intimate and complicated as those of the nervous and vascular systems." (P. 39.)

The signs of *local irritation* are, 1st, an alteration in the proper sensation or action of a part. 2dly. Pain without inflammation. 3dly. Inflammation as a consequence when local irritation is acute and permanent. When this state exists in a high degree, it becomes transferred to the constitution, either immediately (that is, without the medium of inflammation,) or after a short time as a consequence of it. The terminations of local irritation are said to be in resolution, local inflammation, or constitutional irritation: and this last termination our author subdivides into the direct and reflected.

The second section is devoted to the consideration of *constitutional irritation*, and is on every account deserving of particular notice. The causes of this very alarming condition of the system are said to be, 1st, compression, concussion, and all lesions, whether chemical or mechanical; and 2dly, inflammation the result of local injury. Our author, after observing that the symptoms of constitutional irritation produced by severe injuries differ essentially from those set up by inflammation, remarks that, when the irritability of the system is morbid, simple causes of irritation become extraordinary: in fact, it is an extension of the same principle by which we attempt to explain the modifications of inflammation induced by a vitiated state of the secretions.

The next paragraph we give in Mr. Travers' own words:

" Constitutional irritation I consider to be of two kinds, direct and reflected; by which arbitrary distinction I mean to imply, that the first is wholly and immediately derived from the part, commences and is identified with the local mischief, and the constitution has no share in its production. The second, on the contrary, originates in a peculiar morbid state of the constitution to which the injury or inflammation has given birth, or, it may be, previously existing. The first is truly symptomatic, never originating spontaneously, and being immediately induced by the local irritation, is capable of being essentially mitigated or arrested by its removal. The second is occasionally purely idiopathic, and being oftener the cause than the effect of the local action, is seldom influenced by local treatment. In the first, the local appearances are conditions depending on local causes; in the second, they depend on constitutional causes. The symptoms characterising direct constitutional irritation are, in the nervous system, rigor, delirium, convulsion, coma; in the vascular, the fever of phlegmonous, suppurative, ulcerative, and gangrenous inflammation. Those which belong to reflected constitutional irritation are, in the nervous system, epilepsy, tetanus in all its modifications, and other anomalous forms of spasm, mania, &c.; in the vascular system, the fever accompanying scrofulous and carcinomatous inflammation, erysipelas, carbuncle, &c. I deem it no objection to a division of this sort, that the parts are so blended and interwoven as to render the outline here and there obscure, or even imperceptible: it is a circumstance unavoidably resulting from the nature of the subject. Nay, if their boundaries did not mutually encroach upon each other, we might be assured that the division was too artificial to be founded on correct observation, and therefore leading to erroneous views of disease. The extremes must be indicated in order to fix and illustrate the fundamental principles of the distinction; and this is all that is required for useful purposes. In pathology, it would be absurd to expect that a line of demarcation could be laid down with accuracy approaching to that which obtains in the mathematical sciences. Let the justness of the principle be admitted upon which the division is founded, and differences upon the accuracy of the references and details may be easily reconciled. I am quite prepared to admit, that cases are of no uncommon occurrence in which, after an interval, the reflected supervenes upon the direct irritation; which may, therefore, be regarded as examples of mixed irritation, the part and the constitution acting and re-acting alternately upon each other." (P. 47.)

Our author concludes this section with considerations on the influence of contingent circumstances on the production of constitutional irritation from local injury, and which he enumerates in the following order:—1. The texture or organ injured. 2. The kind of injury. 3. Its magnitude. 4. The subject of the injury.

We must leave our readers to reflect on these various causes and their combinations, and proceed to Chapter 3d, entitled *Examples of Direct Irritation*, of which the following causes are enumerated:—1st. Sudden, extreme, and unremitting pain, and certain affections of the mind co-operating with bodily disease. 2d. Injuries and operations of various kinds. 3d. Inflammation, and its termination in suppuration and gangrene. 4th. Exhaustion from hemorrhage or colliquative suppuration; and 5th. Poisons, animal, vegetable, and mineral. Of all these various conditions, Mr. Travers relates numerous illustrations, including cases of sudden death after parturition, severe burns, fractures, dislocations, lacerations, &c.; surgical operations, inflammation succeeding to operations, colliquative suppuration, profuse hemorrhage, &c.

But we pause at the fifth section, which treats of *poisons*, because this includes the principal object of the work itself,—the description of wounds received in dissection; together with the peculiar views entertained by our author on the nature of such wounds, and the causes of the accidents to which they give rise. Mr. Travers commences this section by observing, that some of these cases (namely, of wounds received in dissection,) are to be regarded as apposite and familiar instances of the irritation set up by poison. Many cases, he adds, of rapid inflammation following slight wounds in persons accustomed to handle dead animal substances, *in a state more or less of decomposition*, undoubtedly belong to this class; and he appears to give his sanction to the common phrase, "that the wound is poisoned;" and this is further confirmed in a subsequent part of the same paragraph, in which, although he admits that a predisposed irritable state of the system is almost always present in these instances, and that he has had occasion to witness many alarming cases of diffused inflammation arising from the slightest causes, without any suspicion of animal or other poison being absorbed; yet "the fatal effects of poison taken into the system are but too rapidly developed, *although the previous health has been undisturbed*, and the existing inflammation is insignificant." Our author pursues the argument thus:

"That a puncture with a knee or shoe buckle, or a pen-knife, has now and then proved speedily fatal through the medium of inflammation; and, on the contrary, that persons residing in the country, in full health and habits of exercise, are seldom visited by any troublesome degree of inflammation from these accidents, only corroborates the influence of constitution; which I admit. But it is said many persons escape inflammation who are equally the subjects of these inoculations with those who pay the forfeit of their lives. This is a statement of which it is impossible to ensure the accuracy, and from which, consequently, no fair inference can be drawn. It is a well-known fact, that, if two men cohabit with an impure female, it happens continually that one escapes the disease. Let it be shown that the poison is imbibed by any two individuals, and then we are beyond the reach of contingency, and it will be fair to inquire why one person is destroyed and the other is unharmed." (P. 201.)

To this passage a note is appended, which is adduced as a curious and interesting proof of the difference in effect between an inoculated and simple puncture. Two gentlemen punctured themselves with a needle in opening a body, but one of them received the wound after *wiping the needle to put it by*. The person who was wounded with the dirty needle (Mr. Archer) died, the other had thecal inflammation, with affection of the absorbents; the axillary glands were also acutely inflamed, and the local effects were as strongly marked as in the fatal case. The constitutional symptoms, however, were very slight.

But to continue. Our author's distinction between acute cellular inflammation proceeding from simple injuries, and exciting through that medium a fatal constitutional irritation, and those cases of inflammation arising from the poison of animal matter, is this, that, in this latter case, the inflammation is only a sign, and not the disease; and that the symptoms of constitutional irritation are not depending upon the inflammation, though they may occasionally be aggravated by it. Indeed, our author is disposed to think that the symptoms of local inflammation and constitutional irritation exist in an inverse ratio of severity: this is illustrated by a short case, which we shall insert.

"Mr. Elcock, student of anatomy, slightly punctured his finger in opening the body of a hospital patient, recently dead, about twelve o'clock at noon, and in the evening of the same day (Monday), finding the wound painful, showed it to Mr. Cooper, after his surgical lecture, by whom he was referred to Dr. Haighton, in whose house Mr. E. at that time resided. He applied a poultice to the finger, and took some active aperient medicine. During the night the pain increased to extremity, and symptoms of high constitutional irritation presented themselves on the ensuing morning. No trace of inflammation, however, was apparent, beyond a slight redness of the spot at which the wound had been inflicted, which was a mere puncture. In the evening he was visited by Dr. Babington, in conjunction with Dr. Haighton and Mr. Cooper. Still no local change was to be discovered, but the nervous system was agitated in a most violent and alarming degree, the symptoms nearly resembling the universal excitation of hydrophobia; and in this state he expired at three o'clock on Wednesday morning, within the short period of forty hours from the injury." (P. 203.)

Immediately after the relation of this case, Mr. Travers proceeds to enumerate four different textures subject to inflammation from injury: 1st, the absorbents and their glands; 2d, the veins; 3d, the cellular membrane, the inflammation of which is either phlegmonous or erythematous, erysipelatous or gangrenous; and 4th, the thecæ of the tendons and fasciæ of muscles: all which forms of inflammation, though distinct in their origin, are, he says, subject to complication from their continuance.

Each of the above conditions occupies the attention of the author seriatim, but he dwells more at length upon that affection of the cellular membrane which Dr. Duncan has treated of under the term of diffuse inflammation. He describes it with much minuteness and precision, and expresses his belief that this is a specific inflammation peculiar to poison; he carefully distinguishes it from common phlegmonous inflammation terminating in gangrene, and what he calls gangrenous inflammation, which in fact (if we understand rightly) is only the more speedy effect of a rapid form of common inflammation.

In the description of the effects of inflammation of the aponeurosis, thecæ of tendons and periosteum, we meet with a detail of symptoms so similar to those said to arise from the absorption of animal matter, that we are led at once to perceive the difficulty and intricacy of the subject by the mere enumeration. How extremely difficult the discrimination by mere symptoms must be, may be inferred from the following quotation:

"To recapitulate briefly. Inflammation of the absorbents and their glands, of the vein, the phlegmonous inflammation of the cellular membrane and of the fascia, are all ordinary results of simple irritation, and may be distinct or combined, or consequential, one with another. These comprise all the textures which are the seat of active inflammation from injuries such as those to which I refer. But there is, as I have endeavoured to show, a variety in the modes of inflammation; and, though I am unable to say in what degree the absorbents, and veins, and nerves, and fasciæ, may be subject to be affected by the varieties which I have described as affecting the cellular membrane, it is highly probable that, in some degree, they are.* The open texture of the latter, and its universal distribution as a medium of connexion, enable us better to observe the varieties of which it is the subject. The erythematous, I consider, belongs to a specific irritation, the erysipelatous to a specific state of constitution; the gangrenous is to be referred to age or constitution, if not plainly occasioned by the extent of mechanical disorganisation." (P. 219.)

* "The veins and absorbents have been found to contain pus through an unbroken course of their canals. Such a termination is always fatal. Owing to the defect of adhesive inflammation, no barrier, and of consequence no abscess, is formed. This must be the result of a morbid variety of inflammation; for the disposition to adhesion is stronger in both these orders of vessels, and such deviations are, I believe, the results of specific irritation, as e. g. a poison."

A considerable portion of this section is filled with the details of the cases of Dr. Pett, Mr. Dease, Mr. Newby, Mr. Archer, Mr. Delph, and others, some of which have appeared in this and other Journals; it is therefore with the less regret that we pass them by, as incapable of abridgment. We are compelled to say, however, that we cannot exactly follow our author in the minute distinctions which he has pointed out between some of these cases, which he refers to simple irritation, and others, which he admits as instances of specific absorption. He appears, indeed, to be prepared for this objection, as will appear by the following passage:

"But, it may be asked by those who incline to a contrary opinion, may not a difference in the quality of the poison, or in the susceptibility of the individual, account for the difference in the phenomena which ensue?

"I believe that the difference in quality may explain the greater or less severity, but not the difference of phenomena. If of two individuals injured in dissecting the same subject, the case of one present the symptoms of absorption, and the other not, (as, for example, Mr. Dease and Mr. Egan, Mr. Hersey and Dr. Hennen,) I infer that the one absorbs the poison, and the other escapes it. The cases of Mr. Blyth and Mr. Young, Mr. Delph and Mr. Smartt, dissecting the same subjects, were affected with symptoms precisely similar each to the other. The absorption or non-absorption may be determined by accident, the clean or the foul instrument, &c.; or it may be referred to the second head of the question,—viz. the difference in susceptibility. The frequent exposure of some individuals to this and other poisons with perfect impunity, is a matter of notoriety; and this may explain why the specific disease is not set up, when that which results from simple irritation is: and, in truth, the greater susceptibility of local irritation and inflammation may be a condition coupled with insusceptibility of absorption, and afford in some measure a safeguard to the constitution. Thus, if the poison operate as a chemical irritant upon the part, and this inflame, whatever mischief extends to the system is referable to symptomatic irritation; but if it find a quiet and ready entrance into the system, as by an open door, the system is alarmed first, and the local consequences are displayed at a distance from the seat of injury. To what causes this susceptibility is to be referred, or to what extent it operates, we are ignorant; and whether the nature of the wound, affording a facility for absorption in the one case, and not in the other, be the real explanation: however, the fact is undeniable. But this theory of susceptibility supposes no difference in the phenomena characteristic of absorption, but refers the difference in symptoms to the fact of non-absorption. In severity, the symptoms of both specific and simple wounds undoubtedly vary: those, however, of the former less than of the latter. The appearance of vesicles or pustules, or the contrary,—the affection of the opposite limb or side of the body, or the definitive boundary of the action by the median line,—the rapidity and vehemence with which the symptoms of pain and cerebral disorder are manifested, or the reverse,—indicate different degrees of severity. Compare the cases of Dr. Pett and Mr. Dease with those of Messrs. Delph and Smartt. Whether it be to local circumstances or constitutional peculiarity that some individuals owe their security in situations of exposure, I imagine that, if the poison be once admitted into the circulating system, none are exempt from its effects." (P. 331.)

It may be further asked, says our author, whether, as well as in degree, there may not also be a difference in the kind of poison? by which may be explained the formation of pustules in some, the affection of the glandular or cellular systems in others, &c. &c.

"The following objection to admitting the cases in question to be instances of absorption, appears to me to be decisive: they differ in no important respect from simple injuries with clean instruments, from abrasion and bruise, or inflammation of the cutis even without lesion. From such causes inflamed absorbents and their glands, and continuous cellular inflammation and suppuration, are constantly arising; and there is no evidence to prove that continuous inflammation ever arises from the passage of poisons into the mass of blood. On the other hand, distant, diffuse, and superficial cellular inflammation is certainly not an effect of simple injury; but this is the prevailing characteristic of the local action in the most urgent and fatal cases. Now, although it is in the highest degree probable that the poison varying in intensity should occasion a more or less severe disease, as we see in the cases related, it is as improbable that the character of the specific action should be subject to variety: in a word, it is improbable that the effects of the poison, quasi poison, should be at one time such as we have described, and at another such as are liable to be produced, and frequently are produced, by simple causes of irritation." (P. 335.)

In the following twenty or thirty pages we find our author arguing to make good the distinction he has drawn between the cases of absorption and non-absorption; in the course of which discussion he considers the influence of peculiar irritability, (to which he attaches but little importance *per se,*) and endeavours to account for the anomaly of the first evidence of the introduction of the poison being furnished at a distance from the part injured. Another inquiry also engages his attention in connexion with this subject,—namely, whether any specific contagion be capable of being communicated after death? Now all these questions, and many more, are treated of in a very acute and able manner, but our limits prevent us from doing more than referring to them. The latter question he is disposed to answer in the negative.

The fourth Chapter is a very short one, and consists principally of a recapitulation of the arguments of the former section.

The first section of the fifth Chapter commences with noticing the experiments which have been made by several eminent English and foreign physiologists on the influence of the *brain* over the functions and properties of the parts to which they are distributed; under which head he considers the supremacy of the brain, the superior contractility of the involuntary muscles, illustrated by experiments, the force of habit, and the "inimitable" nature of the nervous function.

The second section treats of *derangements of the nervous system,* both physical and functional. Under this head are considered those instances of failure or suspension of the nervous power, exhibited in many instances of sudden death, or even after days or weeks from the injury, wherein there are no traces of destruction of texture to be observed. This leads to some remarks upon the mode in which such accidents prove fatal; and our author believes that the first impression is made upon the heart. In the course of this discussion, Mr. Travers explains a distinction between direct and indirect affections of function. The direct injury of an organ, he says, affects the functions secondarily; that which is indirect, or sympathetic, acts first upon the function. This he illustrates by several examples; but we are restrained, by want of room, from entering upon this subject.

The sixth Chapter is on the *Pathology and Treatment of direct Constitutional Irritation;* and first of the *state of prostration without reaction.* This condition is pretty generally understood, and therefore, without stopping to consider the explanatory matter, we proceed at once to state that the indications to be fulfilled in these cases are, 1st, to maintain action; 2dly, not to force action. The first requires the incessant observation of the surgeon, since no nurse can be qualified to direct the administration of the stimulus. Purgative medicine should not be given until the circulation is restored and pretty steady. The respondence of the circulating forces to an increased supply of stimulus, must serve as a caution against over-supply.

Excessive reaction produced by over stimulation, is *prostration with excitement.* Mr. Travers presents us in this section with some excellent remarks upon the indiscriminate use of blood-letting after accidents; a mode of practice much abused, but now beginning to be reduced within the bounds of common sense.

A page or two are next devoted to the general treatment of *severe burns,* and the author sums up his opinion thus:—"Upon the whole, the results of the stimulant treatment of burns, which I have more strictly pursued since my attention has been directed to the subject of this essay, have proved highly satisfactory." (P. 465.)

The treatment of prostration with excitement and excessive reaction, consists in applying an evaporating lotion to the shaved head, a large blister to the nape of the neck, and clearing the bowels thoroughly. Medicines taken by the mouth, if not rejected, are usually inert. With regard to opium, Mr. Travers is of opinion that, in the form of enema, it is often highly useful; but, in whatever way it is exhibited, it is only in full doses that it is admissible in these cases. The henbane, as an anti-irritant, is also highly commended.

In the section on *constitutional irritation from inflammation,* our author observes, that inflammation is strong and effective, or the contrary, as it is free from irritation or mixed with it; and further on he insists still more strongly on the distinction between irritation and fever, of which, indeed, the former may be a symptom, and vice versâ; but they are, he repeats, originally and essentially distinct forms of disease.

In the treatment of inflammatory fever ensuing upon complicated injury, we are told that the chief indication is to tranquillise action, without materially diminishing power; and that the want of fever in such cases is the more alarming circumstance of the two. With respect to local applications, also, the following sentence is too important to be passed by: we fear that it is too little attended to by even modern surgeons:

"An indication of equal importance is to indulge to a certain extent the first actions of the part, by an easy position, and light and soothing applications. We should allow for the effects of local, as well as of constitutional reaction; the swelling and tension must not be aggravated and rendered a source of additional irritation, by nice adjustments and contrivances,—undoubtedly calculated to secure the best possible results, provided we could overlook or command the intermediate changes.

"The application of lint, moist or dry, to a fresh wound, quickly becomes an irritant; and adhesive plasters, circular bandages, and splints, applied (as they too often are) while effusion is going on, operate as strictures and ligatures. Plugging a fresh wound to force suppuration, or strapping it to prevent that process, are equally liable to be followed by injurious consequences. The attempt to render complicated wounds simple, by leaving them in the original close dressings for many days in succession, has proved a common, and sometimes fatal, mischief. I have seen more than one case in which the constitutional irritation arising suddenly, and proving fatal in a week from the injury, was distinctly referrible to a diffused erysipelas and gangrene, set up by such local irritation and stricture. A sufficient inspection of the part at intervals of two days, to satisfy an experienced eye of its well doing, may be accomplished, not only without detriment to the process of healing, but with manifest relief to the patient, and is a widely different thing from handling and disturbing the position of parts, against which I would be among the first to protest as uncalled-for and injurious. Penetrating wounds attended with hemorrhage, and comminuted fractures with extensive injury of the soft parts, and in the vicinity of large joints, are especially the cases which present themselves to my mind in offering this remark." (P. 496.)

The fourth section, on *constitutional irritation arising from loss of blood,* we do not make any extracts from, having in a recent Number entered fully into the subject, when reviewing the paper of Dr. Marshall Hall upon this interesting topic; but pass to the next, which treats of *constitutional irritation from the absorption of animal poison,* and here we find our author again passing in review all the cases of this description which had been noticed in a former part of the work; and with the following remarks upon the treatment of this form of the disease, we close our quotations:

"When inflammation is set up sufficient to draw the patient's attention to the injury, the same simple and soothing applications which would be made to any inflamed part, are, in my belief, the most eligible. If much tension be present, emptying the vessels freely by a deep incision with a keen lancet through the line of the wound, and then enveloping the part in a poultice, is a practice which I have commonly employed, and I have thought with advantage.

"For the erythema of the arm or trunk, folds of linen moistened with the diluted liquor plumbi or liquor ammoniæ acetatis, are probably the best applications. Poultices oppress by their weight, irritate when they lose their moisture, and can seldom be borne, however well prepared. If, in protracted cases, the cellular membrane becomes much loaded, incisions are beneficial to quicken the slow and imperfect suppurative action. In Mr. Delph's case, they were undoubtedly advantageous.

"It appears to me that no rules for constitutional treatment to hold in all cases, can possibly be laid down; that we should err equally in uniformly prescribing a stimulant or a depressing treatment for these cases. The cause of irritation, the symptoms, and the habit and state of health, must be referred to, and from the aggregate the inference must be formed. As regards the first, I

feel warranted in saying, that the plan of early support formerly adopted in these cases of animal poison, under the notion, however erroneous, of a rapid putrescency of the fluids, is more consistent and rational than the antiphlogistic, which assimilates them to inflammation. Venesection, it is true, is one mode of relieving congestion; but a more pernicious one could not be devised where the congestion is the obvious result of a sudden and extreme depression of nervous power. This practice may be said to have had a fair trial in the cases of Mr. Dease and Dr. Bell: in neither did it afford sensible relief, nor did the blood assume any character of inflammation.

"Nevertheless, if a case presented itself, bearing the specific local character, in which the acute pain was combined with steady, contracted pulse, and other signs of inflammatory excitement, I should consider the trial of copious blood-letting in the commencement indicated, nay called for; but I never met with such a case; and for pain upon a vacillating, throbbing, or faltering pulse, the experiment would be unwarrantable. Certain symptoms of depression, however, which are more of a moral than a physical cast, are sometimes sufficient to mask the actual energies of the system, as the full and swinging pulse, peculiar to irritation, deceives an inexperienced person with the idea of strength. Where the use of the lancet is prohibited, pain should, if possible, be allayed by opium. It is of itself, as we have seen, a disease capable of proving destructive." (P. 536.)

We have now concluded a very imperfect and compressed account of Mr. Travers' work; and, from the very nature of the subject, as well as from the manner in which it is discussed by the author, it was scarcely possible to do more within the limits of a review. We trust, however, that we have said enough to induce our readers to turn to the volume itself, which contains much interesting matter; as, indeed, may be partly gathered from the outline we have given of it.

---

*Doubts of Hydrophobia, as a specific Disease, to be communicated by the Bite of a Dog; with Experiments on the supposed Virus generated in that Animal, during the Complaint termed Madness.* By Robert White, Surgeon, Brighton. 8vo. pp. 146. London, Knight and Lacey. 1826.

A Mr. White, of Brighton, is fully persuaded that there is no such disease as hydrophobia, nay he has written a book upon the subject, which extends to one hundred and forty pages of letter-press. It is a very weak production, and we should not have thought it necessary to bring it under the notice of our readers but for this reason, that, as it is addressed to those who are "unprejudiced by education or professional dogmas," (p. v.) it is not impossible that patients may occasionally enquire about it, and therefore it is desirable that the practitioner should be acquainted with the character of the work, and the line of argument adopted.

And, before we enter more immediately into the subject, we cannot but sympathise with the author on the serious evils accruing to the once flourishing town of Brighton,—evils which it has generally been thought have arisen from the absence of a certain august personage, but which it appears, from the statements before us, are rather attributable to the supposed presence of mad dogs. " Numberless families thereby, we have no question, have been prevented from residing with us, *to the great pecuniary injury of the settled inhabitants ;*" and again, " for three years has Brighton now been in a sense depopulated by this fear-spreading cause." These and similar passages sufficiently prove that the abolition of hydrophobia is looked upon as very desirable by the " settled inhabitants" of the place.

It is argued that symptoms analogous to those of hydrophobia are sometimes witnessed from wounds, particularly those made by laceration or puncture, under circumstances which preclude all possibility of their having originated in the bite of a rabid animal. This is true,—tetanus sometimes resembles hydrophobia very closely, and hysteria may imitate either. Coma may be brought on by external injury, or by the administration of a narcotic; but does any one argue from this that the affection is identical in both these instances, or that because a man may be rendered comatose by a blow, therefore there is no such thing as coma from a narcotic poison? The idea of a specific poison or virus is further objected to, because we cannot fix a definite time for the supervention of its effects,—" at the end of a few days, a few weeks, a few months, many years, and the same effects are reported to take place from the self-same causes." This is not a fair statement of the case. It is well ascertained that about forty days is the average period which intervenes between the infliction of the bite and the supervention of the symptoms; the cases in which the disease has come on at the end of a " few days," are extremely rare; and there is not one well-authenticated instance on record in which " many years" have intervened, or even *years* at all.* The interval between the inoculation and the supervention of constitutional symptoms

* The only case of this kind entitled to any notice, is one which was communicated by Dr. Bardsley to the Manchester Society, in which twelve years intervened between the bite and the death of the patient. He had for some time laboured under " nervous agitation," and it was obviously one of those cases which have been called spontaneous hydrophobia.—See the cases published by Dr. Whymper in the preceding volume of this Journal.

varies considerably in different animal poisons, as small-pox, scarlatina, and measles; but yet more remarkably in venereal affections, the intervals being scarcely more definite than in hydrophobia itself. When the disease comes, it is asserted, that, " instead of a specific order, we have indescribable incongruity; not a lively picture of complaint, too descriptive to be mistaken, but a pallet, crowded with imposing colours, thrown out without order, disposition, or effect." How entirely at variance with fact is this tirade! There is not in the whole range of nosology a disease marked by more characteristic symptoms than hydrophobia, or one presenting a more " lively picture." We are quite satisfied that this will only be doubted by those who have never witnessed it.

It has long been well known that rabid dogs do not evince any dread of water; this, the author seems to think, is a discovery of Mr. Blaines, and at all events one which must " appear strange" to the believers in hydrophobia,—" for surely," says he, " it will not be for a moment advanced, that a dread of water shall exist to form the very denominative symptom of complaint that a man receives from a brute, when by an authority like Mr. Blaine's it is distinctly denied that such a symptom is present when the brutes themselves are affected!" We would answer that the *denominative* symptom, as he calls it, is frequently absent in man, many patients having a desire to take fluids, and attempting to do so till they are prevented by the spasms which every effort at deglutition brings on; so that the dread of water is frequently merely the result of experience. Secondly, that dogs are frequently unable to swallow liquids; and thirdly, that were it true, as he erroneously supposes, that the symptoms in mad dogs differ so essentially in this respect, it would remain for him to shew that where a disease is communicated from one species of animal to another, the phenomena in the two must necessarily be identical. That this is not the case is proved by the great modification which cow-pock undergoes when introduced into the human system.

Not the least absurd proposition in the volume is, that, granting the existence of a virus, the punctured wound made by a bite would not be favourable for its absorption: it is only necessary to mention this, to show the extent to which the author's judgment is misled by his preconceived opinions. Immediately after this follows the assertion that " a wound from a lancet armed with poison would be subject to no such objections, and yet it has failed in every instance to produce the effects reported to take place from the puncture of the tooth." Now, this is one among numerous instances in which the author shews his ignorance of the subject on which he has taken upon himself to descant. We beg to inform him that Magendie and Breschet have produced rabies in the dog by inoculation with the saliva of a hydrophobic man,—an *experimentum crucis* which outweighs Mr. White's entire volume. The following short quotation will at once shew the manner in which the subject is handled, and justify the severity of our censure. Alluding to the similarity of tetanus to hydrophobia, he says, "May it not have happened, in this mad-dogmatical wonder-loving age, that men have existed who wanted an intention to mark a distinction, when such distinction would interfere with established dogmas, and go far to spoil the trade of mystery?" And continues: "If this disease, this mystifying Bo! catch-ye! *Hydrophobia,* have indeed a claim to our credence, as arising from a specific virus, the claim may be readily shewn. What is this *virus?*—where is it to be found?—and, when procured, will experiment confirm its reputed effects? It either was never found, or experiment has never made that finding known to the world. I would act fairly in argument, but I do not feel myself called upon to dispute (and I assume the strain of argument only from courtesy to those who hold different opinions,) on what has never yet been proved to have existence. I would rather the onus probandi, in that particular, lay on the shoulders of its advocates. I more than doubt, I have, by my conduct, most unequivocally denied that I believe such a *virus* to exist; and I call upon its supporters to prove its existence, before they attempt to explain its nature or attributes."

The inaccuracy of the assertion in the first part of this quotation, with regard to the result of experiments with the poison, we have already shewn: with respect to the conclusion,—that we must prove the existence of the virus before we explain its effects,—we would beg to enquire whether the same does not equally apply to poisons the existence of which no one doubts—except, perhaps, Mr. White. We ask the same questions concerning marsh miasmata which he has done concerning hydrophobia,—"What is this virus? where is it to be found? and, when procured, will experiment confirm its reputed effects?" It is, like the virus of hydrophobia, a poison which we know only by its effects, and, in fact, this is the state of our knowledge with respect to many morbid phenomena.

The chief argument, however, and that on which the author evidently lays most stress, is the fact of a dog, supposed to have been rabid, having bitten six individuals, none of whom at the time the statement was drawn up, had become affected with hydrophobia. It is thus imposingly set forth, "*Particulars of a Case of Madness in a Bitch, by which a Woman, four Children, and the Author, were bitten.*" The story is too long for quotation, occupying no less than twelve closely printed pages; but all that is essential may be very soon told. On the 22d of May, 1825, four children were brought to the author, having been bitten two days before by a dog supposed to be mad; the wounds were freely scarified, and then had oil of turpentine applied to them. Having ascertained where the bitch was, he went to obtain possession of her, and he informs us that there was then "no appearances unusual" about her,—"she seemed well and playful." Indeed, so little did our author apprehend any mischief, that he suffered her to put her fore-feet on his knee, and "snap in seeming puppy friendliness" at his hands. On the 23d "she ate heartily of some raw sheep's liver, but did not drink much of the water." On the 24th Mr. White was roused early in the morning with the information that the dog had escaped, and, in securing her, he was bitten "in two places slightly on the fingers, and then once rather severely" in the right arm; the wounds were not washed for several hours, and no remedies of any kind were applied. Next day (25th) there was a decided alteration in the dog's manner, she tore every thing within her reach, and flew at those who went near her "open-mouthed;" and, in the afternoon of this day, the poor woman to whom the animal had belonged was bitten in the cheek. This woman, having imprudently returned on the 26th to feed the dog, was again bitten "severely in several places on the hands and arm." Much, and naturally, alarmed, she requested Mr. White to apply caustic to the wounds or cut them out; and, as he most unjustifiably refused to do either, she went to another surgeon, who did both. The author adds, in a sneering manner, "that gentleman, however, somehow left untouched the wound in the face, which probably was the woman's fault, who, being neither old nor ill-looking, might not like to have the caustic prey upon her damask cheek. Be this as it may, this wound and one or two other slight touches on the finger escaped Mr. Coleman's eyes, and consequently Mr. Coleman's knife and caustic." On the morning of the 27th the dog was found dead, having taken no water and very little food since the 25th.

Let us now examine the degree and kind of evidence afforded by this case. Four children were bitten by a dog, which two days after, by the author's own shewing, presented

***no unusual appearances,***—which was ***lively and playful,*** and which three days after ***ate heartily, and drank some water.**** Is it usual for rabid dogs to eat and drink, and to be lively and playful? The four children had the wounds freely scarified, and oil of turpentine applied,—means which it is not unreasonable to suppose, in the event of the animal having been rabid, may have tended to prevent the development of the disease in them :—a circumstance which, therefore, destroys all argument deduced from these cases. With regard to the case of the woman, it is attempted to be shewn, that although some of the wounds were excised, and caustic applied, yet others were left untouched. Is it probable that a surgeon being applied to under such circumstances—the author having previously endeavoured "to calm the woman's fears, but without effect;"—is it likely, we ask, that any surgeon, thus urged by the wishes of his patient as well as by his own judgment, should have left the operation incomplete, when any degree of incompleteness must have frustrated the intention altogether? It is not probable,—and therefore we infer that in these other wounds their extreme slightness, (probably without any abrasion of the skin,) induced the surgeon to regard their excision as unnecessary; and we are the more confirmed in this view of the subject from the manner in which even the author has expressed himself. Alluding to the wound in the face, he says, this, and "*one or two* OTHER *slight* TOUCHES *escaped, &c.*" On viewing the matter dispassionately, we think our readers will agree with us, that the author's own case is the only unexceptionable one. But we shall grant, for the sake of argument, that the dog was really rabid, and that all the cases present fair instances of individuals who must have been inoculated with the poison, —if there be any such thing,—and then ask, what is thus proved? Why, that five† persons may be bitten by a mad dog, and all escape the disease. But so far is this from being any thing new, that Dr. J. Hunter in his excellent paper on hydrophobia, gives an instance of *nineteen* persons who were bit by a rabid dog, without one of them taking the disease; —but then it happened that one more was bit, ***and he did take it.*** In every such question, one positive case outweighs an hundred, or any given number, of such as are only negative: we would therefore recommend Mr. White to remember the fable of the pitcher which was carried to the well in safety

* On the 24th she was observed "to bite one or two dogs," but it is not stated that any of them became rabid.

† One of the children was drowned soon after, and therefore cannot be taken into the account.

ninety-nine times, and to desist from the repetition of what he facetiously calls his "*little experiment.*"

If there be no such disease as hydrophobia communicable by a dog, how is it that individuals so frequently become affected with this dreadful malady after having been bitten? How is it that children become hydrophobic who are not even aware of the existence of such a disease, and long after the immediate effects of the fright have passed away? How is it that the lower animals become affected, in whom no consciousness of the impending evil can be supposed to exist even for a moment? We put these questions to Mr. White, the rather that he has chosen for his motto,—

"Felix qui potuit rerum cognoscere causas."

For our own parts, we are of opinion, that, in order to give any value to the arguments of our author or to his experiments, it would be necessary to shew unequivocally that the bitch *was* mad, and the man who suffered her to snap in "puppy friendliness at his fingers" *was not.*

---

*An Account of the Morbid Appearances exhibited on Dissection in various Disorders of the Brain; with Pathological Observations, to which a Comparison of the Symptoms, with the Morbid Changes, has given rise.* By Thomas Mills, m.d. Licentiate of the King and Queen's College of Physicians.—8vo. pp. 239. Dublin: Cumming, and Hodges and M'Arthur. Longman and Co. London. 1826.

This work consists of a set of cases, ranged under the heads of Hydrocephalus, Cephalic Fever, Apoplexy, and Epilepsy. As may be conjectured from the title-page, most of the patients died; and the history of each case is separately given, first with reference to the symptoms and treatment,—next the appearances on dissection,—and, lastly, we have a short comment. Nothing can be imagined less inviting to the reader, or more difficult of analysis.

There is unquestionably a great similarity in the morbid changes found in the heads of those who have died of diseases, which have very different places in our nosological arrangements. This fact seems to have made a strong impression upon the author, who asks, "Shall the links of the great chain which binds cause to effect be here rent asunder?" In a word, the line of argument adopted is this,—because effusion of fluid, thickening of the membranes, or rupture of vessels, are the most frequent appearances in those who have died with cephalic symptoms, therefore, all diseases in which these phenomena present themselves are very closely allied; and even typhous fever may pass into apoplexy, epilepsy, or hydrocephalus. But it appears to us, that before any reasoning of this nature can be admitted, it will be necessary to ascertain how much of the post-mortem appearance is to be regarded as the cause, and how much as the effect, of the disease.

To give our readers a general idea (and we can do no more) of the author's opinions, and the manner in which he has illustrated them, we subjoin two extracts,—the former from the Preface, and the latter being a case selected on account of its brevity.

"Several cases and dissections are here detailed of hydrocephalus, cephalic fever, apoplexy, and epilepsy.

"These are considered distinct and independent diseases, requiring a distinct and peculiar mode of treatment; yet, when we attentively examine the phenomena and course of each,—when we compare them one with the other, and witness the morbid appearances exhibited in all, and the effects of the same remedies, we shall be compelled to acknowledge that these diseases are closely allied, and, in the true spirit of philosophical research, be disposed to allow that the morbid actions which produce effects so alike, cannot in their nature be dissimilar.

"In regard to these disordered actions, various modifications must occur, proceeding from the intensity and variety of the cause,—the constitution, age, sex, previous habits, and diseases of the patient,—the condition of the atmosphere, and other incidental circumstances.

"It is a remarkable fact, that these diseases of the brain pass and repass one into the other:—Epilepsy, for example, often terminates in apoplexy, and apoplexy in epilepsy; cephalic or typhous fever sometimes passes into apoplexy, sometimes into epilepsy, but most frequently into hydrocephalus; and hydrocephalus is often accompanied in its progress by an epileptic or apoplectic paroxysm.

"The phenomena of these diseases likewise merit the serious attention of the reader: in each he finds the prominent symptoms to be headache, delirium, stupor, coma, sense of weight or fulness of the head, vertigo, tinnitus aurium, convulsions, paralysis of the sphincters and other muscles; all which are indicative of disordered actions of the vascular system of the brain, and of a disturbance of the sensorial functions. To these symptoms we may add, a pulse varying in strength, frequency, and regularity, a varying temperature of the skin, and an irregular state of the secretions.

"To these remarkable circumstances I shall subjoin the appearances most commonly discovered in the brain on dissection,—viz. effusion of serum, coagulable lymph, or blood, thickening of the membranes, rupture of blood-vessels, or formation of purulent matter.

"Nor can we pass over in silence the remedies usually employed with a view to the cure of these disorders, which consist, for the most part, in blood-letting, general and topical; in blistering; in the exhibition of aperients, mercurials, antimonials, and sudorifics; and in the use of counter-stimulants.

"Further, in regard to their prevention, we find all practitioners agreed as to the propriety of adopting the same regimen, and of establishing one or more drains in the head or its immediate vicinity.

"I now beg leave to submit these matters to the consideration of the reader, and then I would ask—Can these disorders of the brain be looked upon as distinct and independent, requiring a distinct and peculiar mode of treatment?"

"*A Case of Hydrocephalus, supervening to Measles.*

"July, 1821.—Mrs. B——'s child, æt. sixteen months, Baggot-street; during the last week has laboured under hydrocephalic symptoms: moaning, sighing, vomiting, knitting of the eyebrows, frequent opening and rolling of the lips, præcordial oppression, convulsions, with a pulse varying in strength, frequency, and regularity,—at one time 130. Has been in the country, in consequence of some delicacy of constitution and of cough, occasioned by measles, which appeared in this child about a month ago: the eruption was copious and the fever high, attended by a great degree of languor and oppression. Leeches have been applied to the temples, and blisters to the head and nucha; calomel and aperients have been administered. Dr. Labatt in attendance.

July 1st.—Is able to sit up in bed; sees, hears, and swallows; pupils natural; pulse eighty-six, regular; fæces greenish.

Pulv. ex Cal. et Rheo.

July 2d.—Convulsions, stupor, delirium; pulse 120, irregular and intermitting; face alternately flushed and pale; moaning and sighing; fæces green, yellow, and curdy; pupils contracted.

Vesic$^{m}$. occipiti. En. Tereb.

Cal. c. Opio. Baln. tepid.

"July 3d.—Nearly as yesterday.

"July 4th.—Died this morning.

"*Dissection*, by Mr. Buchanan and Mr. Brady.—Serous effusion is perceptible between the arachnoid membrane and pia mater.

"Considerable turgescence of the vessels on the surface of the brain. The depressions between the convolutions are filled with serous fluid.

"Lateral ventricles distended with a watery fluid, about an ounce of which was collected: a large quantity was lost.

" Gall-bladder distended with dark-coloured bile.

" *Observations.*—It is not unusual to see hydrocephalus supervene to measles.

" Does not this point out the necessity of early depletion in measles, especially in children predisposed to hydrocephalus, with a view to obviate the occurrence of so fatal a malady?

" Is the inflammatory affection of the skin in measles communicated to the lining membrane of the brain?—or does the general excitement of the skin, and the suppression of perspiration, induce congestion and excitement of its vessels?

" Here the symptoms were indicative of hydrocephalus, and are accounted for by the appearances after death." (P. 50.)

Numerous cases more or less similar are detailed, and the above may be regarded as a fair specimen of the manner in which the subjects are treated.

---

*Observations on* M. Laennec's *Method of forming a Diagnosis of the Diseases of the Chest by Means of the Stethoscope, and of Percussion; and upon some Points of the French Practice of Medicine.* By Charles Scudamore, m.d. f.r.s. Member of the College of Physicians in London; Honorary Member of Trinity College, Dublin, of the Medico-Chirurgical Society of Edinburgh, and of the Medical Society of Paris; Member of the Medico-Chirurgical Society of London; Physician in ordinary to his Royal Highness the Prince Leopold of Saxe Coburg, &c. &c.—8vo. pp. 123. London: Longman and Co. 1826.

We sent this little volume to one of those gentlemen to whom we are indebted for occasional assistance: it was returned with the laconic answer—*Ex nihilo nihil fit;* and a perusal of the work has not disposed us to differ much from this estimation of its merits.

The first part relates to the stethoscope, and consists of a few desultory remarks upon the subject, apparently made during a very short visit to Paris; but containing not a single fact or observation which has not been repeatedly mentioned in the various works on Mediate Auscultation, which have recently been published either on the continent or in this country. Our readers, therefore, may relieve themselves from any apprehensions of our again entering upon this discussion. For a full account of the stethoscope, and our opinions regarding it, we beg to refer to our review of Dr. Stoke's work in the preceding volume.

The second part consists of general observations, and embraces a multiplicity of subjects treated in a cursory and rather an unsatisfactory manner. We are sorry to speak thus harshly of the performance of any one, particularly of an author whose former writings hold so respectable a rank.

In order to justify these remarks, therefore, we beg to inform our readers that the volume consists altogether of only 123 pages, in a large type and with wide interstices, and that in this short space the following subjects are discussed, and in the following order:—The stethoscope, p. 21; the buffy coat of the blood, p. 22; the stethoscope, p. 23, 24; the buffy coat of the blood, p. 25; the use of digitalis, p. 27; the stethoscope, p. 28; phthisis pulmonalis, p. 30; œgophonism, p. 36; the different rales, p. 37; disorder of the heart, p. 38; disorder of the aorta, p. 41; action of the heart, p. 42; general conclusions, p. 46; introduction of the general subject, p. 47; hospitals in Paris, p. 49; chemistry and morbid anatomy, p. 52; use of calomel, p. 53; use of lavemens, p. 55; use of leeches, p. 59; cupping, p. 61; principles of bleeding, p. 64; French practice of medicine, p. 63; la medecine expectante, p. 64; principles of practice, p. 65; doctrines of Broussais, p. 66; treatment of enteritis, p. 75; gout, p. 76; rheumatism, p. 77; tisanes, p. 78; case in regimen, p. 79; cutaneous diseases, p. 80; tartar emetic, p. 82; sulphate of quinine, p. 88; acetate of morphine, p. 91; black drop, p. 92; hydrocyanic acid, p. 93; nux vomica, p. 95; strychnine and brucine, p. 99; emetine and veratrine, p. 101; general observations, p. 103; water of the Seine, p. 106; case with the stethoscope, p. 109; pathological observations, p. 118; conclusion, p. 123.

Such are the headings of the pages, as given by the author himself, and it is obvious that a volume so constituted bids defiance to any thing like a general analysis. Such of the observations on the subjects above enumerated as appear worthy of notice, we shall take an opportunity of inserting in our COLLECTANEA, under their various departments.

---

*Répertoire général d'Anatomie et de Physiologie, Pathologiques, et de Clinique Chirurgicale; ou, Recueil de Mémoires et d'Observations sur la Chirurgie, et sur l'Anatomie et la Physiologie, considerées dans les tissus sains et les tissus malades.* Numero 1, tome 1; trimestre de 1826.—Paris. To be continued quarterly; with Plates in a separate Atlas.

FROM the well-known celebrity of the contributors, who are understood to be the principal supporters of this work, it may be reasonably imagined that much interesting information will be contained in it. We intend, therefore, to give an abstract of the succeeding Numbers,—at least so long as they continue to answer our expectations. Such articles as we may not deem interesting to our readers in general, we shall pass over without any formal notice, in order that we may afford space for the consideration of the more valuable communications.

The first article, for instance, which arrests our attention can be but of local interest, and will be therefore briefly dismissed. It is a Report made to the Royal Academy of Sciences by M. Dupuytren upon a Memoir of M. Costa, entitled, *General Considerations on the Epidemic which ravaged Barcelona in* 1821, *and upon the Means adopted by Government to prevent its Extension.* The committee appear to have had considerable difficulty in coming to any conclusion, in consequence, not only of the conflicting hypotheses, but likewise of the contradictory facts they had to examine.

M. Costa has adopted the fundamental axiom of the theory of infection, "that the yellow fever can never be transmitted beyond the focus of infection, and that the disease vanishes in the focus in which it originated." We cannot venture to enter upon the oft-disputed, and yet unsettled, question of the contagious or non-contagious nature of the yellow fever. The opinion of the committee adds nothing to our knowledge. "They conceive that, in the present state of science, it is impossible to determine whether the yellow fever is or is not contagious in every case." We must hazard one recommendation to those who enter the field of discussion upon this highly important subject,—that they will first state the precise definition they apply to the terms "contagion" and "infection." The contagion of one writer is the infection of another; and it has not unfrequently happened, from the want of the preliminary arrangement which we advise, that, after a very warm contest, it has been discovered that no other difference of opinion existed than could be removed by a clear understanding of the import of the terms respectively employed by the philanthropic opponents.

Upon the vitally important question of the necessity of guarding against the extension of certain diseases, by lazarettos, quarantines, and purifications of every kind, the committee very rationally observe, "that the means pursued for ages, in consequence of the conviction of the contagious nature of certain diseases, ought not to be abrogated until it shall have been demonstratively proved that such maladies are not contagious. So far from this proposition being firmly established, it is still warmly contested. We cannot, therefore, agree with the author in his opinion of the propriety of abolishing the means hitherto had recourse to. They ought undoubtedly to be continued, until the fallacy of the doctrine which has led to their adoption be satisfactorily proved."

Our readers are doubtless aware that the non-contagionists deprecate the employment of the means which are usually adopted, to prevent the spreading of diseases commonly reputed to be contagious. They, tell us, on the contrary, that the more we scatter the patients the better; and that particular receptacles for the sick, as well as quarantines, &c. are useless, and even dangerous.

The next paper, *Upon a New Species of Extra-Uterine Pregnancy*, by M. Breschet, was published in the last volume of the Medical and Chirurgical Transactions; we shall, therefore, be brief in our notice of it. But three species of extra-uterine pregnancy have yet been admitted, according to Breschet: 1st, abdominal pregnancy, *graviditas abdominalis;* 2d, pregnancy of the Fallopian tubes, *graviditas tubaria;* and 3d, ovarial pregnancy, *graviditas ovaria.* M. Breschet mentions a fourth deviation from the usual course of nature, and proposes to designate it by the name of "pregnancy in the substance of the uterus," *graviditas in uteri substantia.* Seven cases of this species of extra-uterine pregnancy are mentioned by the author, and are illustrated by very neatly executed plates.

The difficulty of accounting for these anomalous cases, is confessed by M. Breschet; and he does not flatter himself that the suggestions he has hazarded upon the subject are conclusive.

A paper of M. Lobstein, *Sur la Kirronose,* is the next in succession. The term "kirronose," is employed to signify a disease of the embryo and fœtus, in which the serous membranes are tinged of a beautiful golden yellow colour. This affection differs from common jaundice, inasmuch as it does not attack the parenchymatous cellular tissue of organs, or the skin, the ordinary seats of icterus. The first time M. Lobstein discovered this disease, was in two embryos of five months. The peritoneum was very highly coloured; that part of the membrane lining the posterior surface of the abdominal parietes was of the deepest yellow. Independent of this appearance, the viscera were in a natural state. There was no effusion contained in the peritoneal sac. The same phenomena were observed in a second embryo. The peritoneal lining of the abdominal muscles was in this case most highly coloured, together with the omentum and the external tunic of the liver. The pleura costalis, the diaphragmatic and pulmonic pleuræ, the pericardium, and the surface of the heart, were also yellow.

Within the cranium, the same appearance was seen upon the dura mater and arachnoid membrane. The thyroid gland, and the adjoining parts, were equally tinctured with the same colour.

Other instances of a similar nature are mentioned by the author. He was at first of opinion that the yellow colour of these embryos was to be found only in the serous membranes, until, upon further investigation, the brain and spinal marrow were examined, and the same appearance was seen in them. The colour of the spinal cord was particularly deep, not only upon the external parts and the enveloping membranes, but even in the interior of its substance. By the aid of a microscope, it was perceived that the cord was composed of small particles, of a citron colour, mingled with a white pulpy matter, as if a fine yellow powder had been incorporated with a soft and semi-transparent jelly.

M. Lobstein found, upon extending his researches still further, that the two yellow bands, or lines, which he had observed in three embryos affected with "kirronose," upon the sides of the vertebral column, were the great sympathetic nerves; the natural colour of which had been changed into a citron yellow, which was also visible in their internal structure.

The yellowness of the serous membranes and of the nervous pulp, is not the effect of any colouring matter foreign to the parts; for it is impossible to deprive them of this tint, either by ablution or infusion in water or alcohol. Embryos preserved for seventeen years in spirit of wine, still retain the colour they did when fresh and recently dissected. It would appear, therefore, that this colouring matter is inherent in their structure, and that it forms a constituent part of the tissues affected. M. Lobstein professes his inability to determine the causes to which this disease ought to be referred. He has never seen it except in the embryo and fœtus.

We are promised, in a succeeding Number, a paper from M. Breschet, upon the Jaundice of Infants, in which the differences existing between that complaint and the kirronose of M. Lobstein will be pointed out.

Upon this subject we may observe, that a yellow hue on the surface of infants is not necessarily a symptom of jaundice. Cullen and other writers have stated, that such a discoloration may also be the result of a peculiar yellowness of the serum of the blood, unconnected with the bile, analogous to the golden tint which we so frequently find diffusing itself over the surface of a contusion, when the finer and more limpid parts of the effused fluid have been carried off, and the colouring matter of the serum that still remains behind is hereby become more concentrated.*

A Memoir by M. Andral, fils, *upon the Anatomical Appearances of Chronic Gastritis,* next claims our attention. The French and German physicians, it is known, have been very zealous in their researches upon this subject. Inflammation of the mucous membranes is thought by some of the continental sçavans to be the root from whence springs most, if not all, the diseases to which mortality is obnoxious. The object of the present paper is rather to discuss the alterations suffered by the parts from disease, than to estimate the frequency of the affection. The morbid appearances presented in subjects who have laboured under chronic inflammation of the stomach, are very accurately detailed, although we are not aware that much is added by the author to the information we previously possessed upon the subject. M. Andral appears particularly anxious, at the commencement of his paper, to show that in many cases, where the mucous membrane is found upon dissection of a natural appearance, but where the subjacent parts have undergone some morbid alteration, the mucous membranes were still the parts originally affected. In support of the probability of this opinion, many analogical facts are adduced. We believe it is not doubted that disease may commence in the mucous membranes,—that it may subsequently extend to other contiguous parts; and that the structure originally attacked may recover its healthy condition, in proportion as the diseased action under which it primarily laboured may be more or less completely transferred to other parts.

The dark appearance of the internal surface of the stomach, which, upon the examination of bodies, is so frequently the subject of discussion, is considered by M. Andral as the necessary consequence of the diminished velocity of the circulation in the capillaries of an inflamed part. It has been demonstrated by Hunter, that, whenever the arterial blood is arrested, or simply retarded in its course, it assumes the colour of venous blood. The experiments of Dr. Wilson Philip and others prove that such a retardation of the arterial blood during inflammation does exist, and hence the explanation of the altered colour of the internal tunic of the stomach.

It occasionally happens, that chronic inflammation of the stomach imparts to the mucous membrane a white milky appearance, very different from its natural colour. M. Andral

* Vide Good's Study of Medicine, vol. i. p. 404, and vol. v. p. 698, 2d edition.

has always found this colour combined with certain other alterations which more unequivocally announced the existence of inflammation, such as a thickened and hardened state of the membrane. Induration of the mucous membrane of the stomach is one of the best anatomical characters by means of which chronic gastritis may be distinguished from the acute species. The same observation will apply to all the membranous and parenchymatous tissues. Acute may frequently produce the same appearances as chronic inflammation. Thus, for example, in the mucous membranes, a morbid softening may be observed, whether the inflammatory action has been rapid or slow. An indurated condition of the part, on the contrary, belongs exclusively to chronic inflammation, and may be either partial or general.

In a recent number we have given some cases, from the German writers, of softening and total destruction of the mucous coats of the stomach. Such instances are very common, particularly in children. It would appear from the observations of M. Louis, who has paid much attention to this subject, that this preternatural softening of the mucous membrane of the stomach is frequently found in phthisical patients.

It is worthy of observation, that in most cases this morbid softening of the stomach is detected at the cardiac extremity of the organ. Inflammation of the pyloric end of the stomach, on the contrary, generally terminates in induration of the mucous membrane and the subjacent tissues. It is probable that this distinction in the effects produced by disease, depends upon some difference in the texture of the pyloric and cardiac extremities of the stomach. It is curious that inflammation appears disposed to produce one particular kind of alteration in preference to another, not only in the different tissues, but even in different portions of the same tissue. For example, acute gastritis rarely terminates by ulceration, whilst nothing is more common than that effect in acute inflammation of the intestines. It has been a subject of frequent discussion, whether the softening which is so frequently detected upon the internal surface of the stomach is invariably the effect of inflammation. M. Andral is induced, from his experience, to answer this question in the affirmative. His researches have been extensive upon this subject, and we receive his opinion with much deference; at the same time we would observe, that a softened and pulpy state of the mucous lining of the stomach has frequently been presented to our view in the bodies of infants who have exhibited during life no satisfactory marks of gastric inflammation. We agree perfectly with M. Andral that it is not just to infer that inflammation has not existed in a part because it is found of a pale white colour. In many parts we cannot deny the existence of inflammation although no redness is visible. Such is the case with serous membranes that secrete pus, and which still generally preserve their natural colour. They frequently present no blush of redness, although they are much softened. It cannot be denied that the induration of the cellular tissue around old ulcers, is a product of inflammation; it is, however, in most cases, of a perfectly white colour. The softening of the transparent cornea is frequently neither preceded nor accompanied by redness, although this alteration in the texture of the part is undoubtedly the effect of inflammation.

In a succeeding paper M. Andral promises to examine the morbid alterations produced upon the other tunics of the stomach.

A short paper follows, *Upon some Pathological Conditions of the Cellular Tissue, situated under the Mucous, Serous, and Cutaneous Systems*, by M. Dalmas, fils; and also a description, by M. Breschet, of *A Congenital Malformation of the Envelopes of the Heart.* The former is not of much interest, and the latter could not be understood without the engraving which accompanies it.

In a paper of M. Lauth, fils, upon the yet doubtful subject of the *Connection of the Placenta with the Uterus*, it is suggested "that the union of the placenta with the uterus is effected by means of vessels which are not sanguineous, and which present all the characters of lymphatics, that the function performed is a true act of absorption which cannot take place by means of veins, because venous absorption, if it does exist, takes place by transudation, and that consequently the function must be performed by the vessels described, because none but lymphatic vessels would be capable of modifying the blood of the mother, so as to accommodate it to the wants of the fœtus; and lastly, that the placenta appears to fulfil in the fœtus the functions which are subsequently to be executed by the intestinal canal, rather than those of the lungs as has hitherto been supposed."

The Number contains, besides, a set of *Cases of Lithotomy*, by MM. Dupuytren, Breschet, and Sanson;—these we shall take a future opportunity of laying before our readers.

